# Characterization of giant endocrine cells in the fundic stomach of African catfish *(Clarias gariepinus)* demonstrated by histochemical, immunohistochemical and ultrastructure microscopy methods suggesting their role in immunity

**DOI:** 10.1186/s12917-024-04237-y

**Published:** 2024-09-14

**Authors:** Hanan H. Abd-El-Hafeez, Sulaiman Mohammed Alnasser, Zyad M. Baker, Mohamed Aref, Mohamed A.M. Alsafy, Samir A.A. El-Gendy, Eman Zahran, Hams Mohamed M. A., Ali H. Alghamdi, Mahmoud Osman Khalifa, Basma M. Kamal, Fawzyah A. Alghamdi, Soha A. Soliman, Diaa Massoud

**Affiliations:** 1 Department of Cell and Tissues, Faculty of Veterinary Medicine, Assiut University, Assiut, 71526 Egypt; 2 Department of Pharmacology and Toxicology, College of Pharmacy, Qassim University, Qassim, 51452 Saudi Arabia; 3 Faculty of Medicine, Assiut University, Assiut, Egypt; 4 Department of Anatomy and Embryology, Faculty of Veterinary Medicine, Zagazig University, Zagazig, 44519 Egypt; 5 Anatomy and Embryology Department, Faculty of Veterinary Medicine, Alexandria University, Abis 10th, P.O. 21944, Alexandria, Egypt; 6 Department of Aquatic Animal Medicine, Faculty of Veterinary Medicine, Mansoura University, Mansoura, Egypt; 7 Department of Microbiology, Faculty of Veterinary Medicine, South Valley University, Qena, 83523 Egypt; 8 Department of Biology, Faculty of Science, Al-Baha University, Alaqiq, Saudi Arabia; 9 Department of Anatomy and Embryology, Faculty of Veterinary Medicine, Aswan University, Aswan, Egypt; 10 Department of Anatomy and Embryology, Faculty of Veterinary Medicine, University of Sadat City, Sadat City, 6010230 Egypt; 11 Department of Biological Science, College of Science, University of Jeddah, P.O. Box 80327, 21589 Jeddah, Saudi Arabia; 12 Department of Histology, Faculty of Veterinary Medicine, South Valley University, Qena, Egypt; 13 Department of Zoology, Faculty of Science, Fayoum University, Fayoum, Egypt

**Keywords:** Catfish, Cellular-mediated reaction, CD21, CD3, CD68, Histochemical, TEM

## Abstract

Endocrine cells in the fundic stomach of *Clarias gariepinus* were characterized in this work using transmission electron microscopy, immunohistochemistry, and histochemistry. Performic acid mixed with alcian blue pH2.5 and silver stain were among the histochemical stains used for endocrine cells. Endocrine cells can be found in the epithelium, lamina propria, submucosa, muscular layer, serosa, and the area between the stomach glands. Endocrine cells with one or more nuclei were found. Endocrine cells were studied using CD3, CD21, and CD68 in an immunohistochemistry analysis. The expression of the lymphocyte marker CD3 by endocrine cells is remarkable. In addition, they had a strong immunological response to CD21 and CD68, which are characteristics of phagocytic cells. Granules of varied sizes and electron densities are packed densely into the cytoplasm of the cells, as seen by transmission electron microscopy. We propose that endocrine cells play a crucial role in immune defense. The role of endocrine cells in the gut’s immune system is an area that needs further investigation.

**Supplementary Information:**

The online version contains supplementary material available at 10.1186/s12917-024-04237-y.

## Introduction

The endocrine system is a complex and tightly controlled network of either endocrine glands or individual endocrine cells. The secretion of hormones regulates and supports various physiological processes, including maturation, reproduction, metabolism, and the generation of energy [1].

The glands release hormones directly into interstitial spaces rather than using a duct system to enter the bloodstream. The primary endocrine glands include the adrenal glands, thyroid, parathyroid, hypothalamus, ovaries, testicles, pineal gland, and pituitary gland. The hypothalamus and pituitary gland are examples of neuroendocrine organs [2].

Endocrine cells, also known as neuroendocrine cells and endocrinocytes, are components of the diffuse endocrine system. When the nervous system activates, these specific epithelial cells secrete hormones or peptides. The epithelial surfaces of the respiratory, digestive, and reproductive tracts contain neuroendocrine cells, either individually or in groups [3].

Diffuse neuroendocrine cells play a crucial role in regulating various physiological processes in fish by producing and releasing a wide array of biologically active substances, including hormones and neurotransmitters. These cells, which originate from the neuroectoderm and endoderm, are specialized in taking up amino acid precursors and decarboxylating them, a process known as amino precursor uptake and decarboxylation (APUD) [4]. In fish, the hypothalamo-hypophysial neurosecretory system (HHNS) is integral to initiating energy-consuming behaviors such as migration and spawning, and subsequently transitioning the body to energy-saving metabolism post-spawning. This system’s regulation is crucial for enhancing fish reproduction efficiency, as demonstrated by the development of pituitary preparations that significantly improve fish maturation and reproductive outcomes [5]. Additionally, enteroendocrine cells (EECs) in the intestinal epithelium of fish respond to ingested nutrients and microbial products by releasing over 30 different hormones and neurotransmitters, which influence metabolic functions such as glucose metabolism, satiety, and gut motility. These EECs are divided into subtypes based on their predominant hormone, allowing for fine-tuned metabolic control in response to various stimuli. Research using zebrafish as a model system has identified multiple EEC subtypes, revealing evolutionary conservation in their roles and providing insights into their developmental programs and physiological adaptations [6]. Collectively, these neuroendocrine cells and systems underscore the complex and multifaceted nature of physiological regulation in fish, highlighting their importance in maintaining homeostasis and optimizing reproductive success.

Endocrine cells in the gut release multiple signaling molecules. Although the signal molecules are technically termed hormones due to their ability to enter the bloodstream, they also exert substantial paracrine effects on a local level. The release of hormones from endocrine cells can be regulated by a variety of factors. Various factors can regulate the release of hormones from endocrine cells. Some of these factors include what we eat, mechanical distortion, other hormones and paracrine, and the nerves that supply the mucosa with nerve impulses. Cholecystokinin (CCK), serotonin, tachykinins, and vasoactive intestinal polypeptide (VIP) are only a few of the gut hormones that have a molecular structure with neurotransmitters in the same species. On the other hand, endocrine cells are the exclusive source of insulin, glucagon, and gastrin, among others [7] .

The presence of giant neuroendocrine cells is currently known in these animal categories: fishes, amphibians, reptiles, birds, and mammals. Therefore, it seems that giant neuroendocrine cells are widely distributed in living organisms. In addition to these animal categories, giant neuroendocrine cells were observed in the octopus (i.e., cephalopod mollusk) [8].

It is important to note, however, that giant neuroendocrine cells are not necessarily present in all genera of these categories. For instance, giant neuroendocrine cells are absent in the bass, sunfish, pike, mullet, shark, and stingray, although they have been examined in various species of these genera. Some amphibia (X. laevis, X. tropicalis), birds (quail), and reptiles (alligator) were also examined for giant neuroendocrine cells, but they are not present. In mammals, giant neuroendocrine cells were identified by light microscopy and electron microscopy in the rats, guinea pigs, rabbits, and human species, whereas they are not present in the cats, dogs, monkeys, and pigs. In summary, it is considered that the occurrence of giant neuroendocrine cells varies among different genera, even within the same class of vertebrates [9–11].

Giant neuroendocrine cells, also referred to as giant cells, were first described in 1962 as large neurons (~ 20–70 μm), containing large, eosinophilic cell bodies, and large basal crescents that lead to superficial cells of various sizes. There are some peculiar features of these cells, as most other neuroendocrine cells and neurons, in general, are small in size. The first occurrence of giant neuroendocrine cells was in pleuronectiform fishes. Since then, they have been documented in a variety of species including other teleosts, elasmobranchs, anuran and urodeles frogs, reptiles, aves, and mammals. However, there are no published reports of giant neuroendocrine cells in aspidobranchs, catostomiformes, petromyzontiformes, hind brain hagfish, and lung fishes. There are also no reports on rat giant neuroendocrine cells, raising questions about their functional significance and phylogenetic distribution, among other subjects [12, 13]. Most mammalian and non-mammalian tetrapods, including frogs, turtles, chickens, and rats, have been found to have giant neuroendocrine cells (60 to 100 μm in diameter) in the preoptic areas (POA) and hypothalami. In the rabbit, the size of giant neuroendocrine cells ranges from 60 to 85 μm in diameter. However, in fish and amphibians, which have giant neuroendocrine cells, the cell diameter is only about 15 to 30 μm. Therefore, giant neuroendocrine cells have a diameter of 60 μm or more in mammals and non-mammalian tetrapods, while they have a diameter of 30 μm or less in fish and amphibians, thereby constituting major differences in morphology and size between fishes and tetrapods [14, 15]. 

The current study described the morphology, histochemical, immunohistochemical, and transmission electron microscopy properties of endocrine cells in the fundic stomach of *Clarias gariepinus.* We employed general and histochemical stains for endocrine cells, immunohistochemical characteristics of endocrine cells were examined using CD3, CD21, and CD68. We chose a marker to document the immunological function of endocrine cells.

The role of endocrine cells in the gut’s immune system is an area that needs further investigation.

## Materials and methods

### Sample collections

Ten catfish were taken from the Nile River by the Assiut Government. Each one weighed 250 to 500 g and was 35 to 45 centimeters long. We purchased live fish samples randomly and commercially from fishermen. The samples were taken in a plastic aquarium to the histological lab in the cell and tissues department of the Faculty of Veterinary Medicine at Assiut University in Egypt for further study.

### Examination with a light microscope

The fundic stomach was the focus of the current study. We used five samples for a light microscopic investigation and prepared the remaining five samples for an ultrathin investigation. We anesthetized the fish using 150 mg/L MS-222^®^ (Sigma-Aldrich Corp., St. Louis, MO, USA) [5] and sacrificed them to collect their stomachs for histological examination. Following the sacrifice of each fish, we removed their stomachs, cut them to separate them, and preserved them in a fixative solution for 24 h.

**Method figure demonstrating the fundic stomach after extraction from fish**: A and B showing external configuration (dorsal and ventral) of catfish. C and D: topography of the stomach (S), liver (L), and intestine (I) can be seen after the abdomen has been cut opened. E and F: Fixed gut samples that showing the fundic part (arrow). G and H show both longitudinal and cross-sectional views of the fundic part.

### Processing of the paraffin-embedding method

The processes for paraffin embedding were based on those used by Solimanet al. [16] and Suvarna et al., [17]. First, we used different levels of ethanol alcohol (70%, 80%, 90%, and 100%) to dry out the set samples. Finally, we used xylene to clear the samples. We then covered the samples with paraplast (MilliporeSigma, St. Louis, MO, USA). We used a Leica RM2125 microtome (Leica Microsystems, Wetzlar, Germany) to cut serial 5-micrometer longitudinal and cross sections. We kept the sections dry in an incubator at 40 °C. We stained the sections for general histological examination using hematoxylin and eosin, performic acid mixed with alcian blue (pH 2.5), and silver impregnation, following the staining procedures described by Suvarna et al. [17].

### Resin-embedded samples ready for ultrathin -sections

The resin-embedding method was used after Soliman et al., [18] and Karnovsky’s fixative. We carefully removed the samples and cut them to exact lengths of 2.0 to 3.0 mm. We applied Karnovsky fixative to the sample overnight at a temperature of 4 °C. We post-fixed the samples with osmium tetroxide, washed and dried them, and then filled them with a pure resin/alcohol mixture. We then embedded them in resin and crystallized them in a 60 °C oven. We used propylene oxide from Merck in Darmstadt, Germany, for 30 min to fix the resin. After that, we used a 1:1 mix of epoxy glue and propylene oxide for about 30 min. Finally, we used the epoxy glue mix for three hours. We mixed 12 mL of dodecenylsuccinic anhydride (DSAA) with 5 mL of Araldite (Huntsman Advanced Materials, The Woodlands, TX, USA) and 5 mL of EMbed 812 (Polysciences Europe GmbH, Eppelheim, Germany) to create the epoxy resin mixture. We added the samples to the epoxy resin mixture and heated it to 60 °C. Next, we added an activator (2,4,6-Tris[dimethylaminomethyl]phenol; 1.5%) to the mixture to initiate the polymerization of the samples. We kept the blocks at different temperatures for three days. We kept the first block at 60 °C, the second block at 70 °C, and the third block at 75 °C. We used a Reichert-Jung Ultracut E ultramicrotome (made by Leica Microsystems) to cut pieces that were about 1 μm thick. After that, we colored the pieces with toluidine blue (Suvarna et al., 2013). We attached a Canon PowerShot A95 digital camera to a Leitz Dialux 20 lens and used it to view the stained sections.

### Immunohistochemical procedures for CD3, CD68, and Cd21

The procedure was done according to Abdo et al. [19]. and Abd EL-Hafeez et al. [20], method. To identify CD3, CD68, and Cd21 in two steps, we used a Dako EN Vision + Single Reagent (HRP. Mouse: Agilent Technologies, Inc., Santa Clara, CA, USA).

5 μm thick paraffin slices were taken out of the wax, rehydrated, and washed three times with PBS (pH 7.4) for five minutes each time. We treated the slides with a mixture of methanol and drops of 3% hydrogen peroxide. We then allowed them to dry at room temperature for 20 min. After that, the slides were run over with water for another 10 min to stop endogenous peroxidase from working. To get the antigen out of the slides, we put them in a sodium citrate solution with a pH of 6.0 (see Table 1) and heated them in a bath of tap water for 20 min at 95–98 °C. After that, we let the slides cool for 20 min at room temperature. After that, we washed the slides three times with PBS (pH 7.4) for five minutes each time. To keep the sections from getting background stains, we put drops of Dako Protein Block (Agilent Technologies, Inc.) on each one and let them sit at room temperature for 5 min. We used the main antibody on the sections, as indicated in Table 2. After the incubation period, we put the secondary antibody on the slides and left them there for 30 min at room temperature. We then washed them three times with PBS (pH 7.4) for five minutes each. We then treated the slides again with DAB and substrate-chromogen for 5 to 10 min at room temperature. We used Harris hematoxylin for 30 s to cover up the colors in the pieces.The pieces were cleaned with xylene, coated with DPX, and dehydrated twice, once for five minutes in 90% ethanol and once in 100% ethanol. We used a Leitz Dialux 20 lens and a Canon PowerShot A95 digital camera to examine the immunohistochemical staining. To make negative control samples, we used a different version of the method that didn’t involve using primary.

**Table 1:** Components of the Fixative

Fixative	Components	Amount
Bouin’s solution	Picric acid saturated aqueous solution	750 mL
	40% formaldehyde	250 mL
	Glacial acetic acid	50 mL
Karnovsky fixative	Paraformaldehyde, 25% freshly prepared	10 mL
	Glutaraldehyde 50%	10 mL
	Na-phosphate buffer (0.1 M, pH 7.4)	50 mL
	Distilled water	30 mL
Na-phosphate buffer (0.1 M, pH 7.4)	Solution A	
	Na_2_HPO_4_·2H_2_O	17.02 g
	Distilled water	600 mL
	Solution B	
	NaH_2_PO_4_·H_2_	6 g
	Distilled water	200 mL
	Using solution	
	Solution A	580 mL
	Solution B	219 mL
Citrate buffer (pH 6.0)	Solution A	
	Citrate C_6_H_8_O_7_·H_2_O	21 g
	Distilled water	1 L
	Solution B	
	Sodium citrate Na_3_C_6_H_5_O_7_·2H_2_O	29.41 g
	Distilled water	1 L
	Using solution	
	Solution A	9 mL
	Solution B	41 mL
	Distilled water	Add 500 mL

**Table 2:** Identity, sources, and working dilution of antibodies used in the present immunohistochemical analysis

Primary antibody	Supplier	Origin	Dilution	Incubation	Antigen retrieval	Biotinylated secondary antibody
CD68 (Macrophage Marker) Ab-3 (Clone KP1)	Mouse Anti-CD68 (Thermo Fisher Scientific Lab Vision Corporation, Fremont, USA)	Mouse Monoclonal Antibody Cat. #MS-397-R7	1:100	Over night	boiling in citrate buffer (pH 6.0), 20 min	Goat anti-rabbit secondary antibody (Cat. no. K4003, EN Vision + TM System Horseradish Peroxidase Labelled Polymer; Dako). Ready to use (30 min at room temperature)
Anti-CD3 antibody [CD3-12]	(ab11089), Abcam		1:100	Over night	boiling in citrate buffer (pH 6.0), 20 min	Goat anti-rabbit secondary antibody (Cat. no. K4003, EN Vision + TM System Horseradish Peroxidase Labelled Polymer; Dako). Ready to use (30 min at room temperature)
Ant i- CD21 antibody	(Abcam)	Rabbit monoclonal [SP186](ab227662)	1:100	Over night	boiling in citrate buffer (pH 6.0), 20 min	Goat anti-rabbit secondary antibody (Cat. no. K4003, EN Vision + TM System Horseradish Peroxidase Labelled Polymer; Dako). Ready to use (30 min at room temperature)

### Digital coloring of transmission images

by using the Photoshop program to colour the transmission images.

### CMEIAS segmentation (all negative figures were presented in the supplementary file)

We made negative images with CMEIAS Segmentation, a free, improved computer method for working with color photos that separates the important item in the foreground from the background. Following are the steps: Open the picture in CMEIAS color segmentation. From the menu, choose “process” and then “negative image.” [16, 20–24].

## Results

Endocrine cells in the fundic stomach were studied for their shape, histochemical and immunohistochemical characteristics. Performic acid mixed with alcian blue pH2.5 and silver stain were among the specific and generic histochemical stains utilized for endocrine cells.

**Fig. 1:**
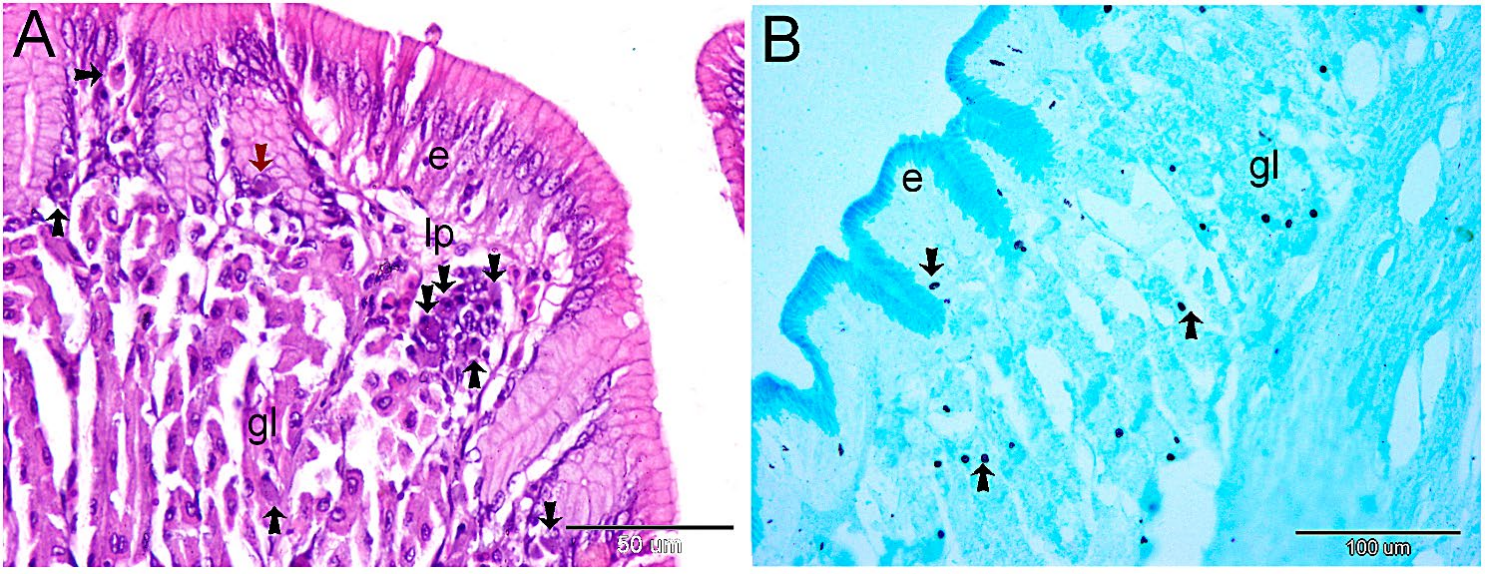
The use of H&E and performic acid combined with alcian blue ph. 2.5 for the histological identification of endocrine cells. **A** and **B** are the results of H&E staining on a paraffin section of the fundic stomach. The epithelium (e), lamina propria (lp), and gastric gland interstitial cells (gl) are all sites of endocrine cells. The red arrow indicates a binucleated endocrine cell. (**B**) Performic acid staining of endocrine cells is positive. The epithelium and the stomach gland (gl) are where you can find them

Epithelial cells, lamina propria cells, and stomach gland interstitial cells that contain endocrine hormones. Endocrine cells with two nuclei are found beneath the epithelium (Fig. 1A). The combination of performic acid and alcian blue, pH 2.5, gave a positive stain to endocrine cells. ig. 1B illustrates their location between the stomach gland and the epithelium.

**Fig. 2:**
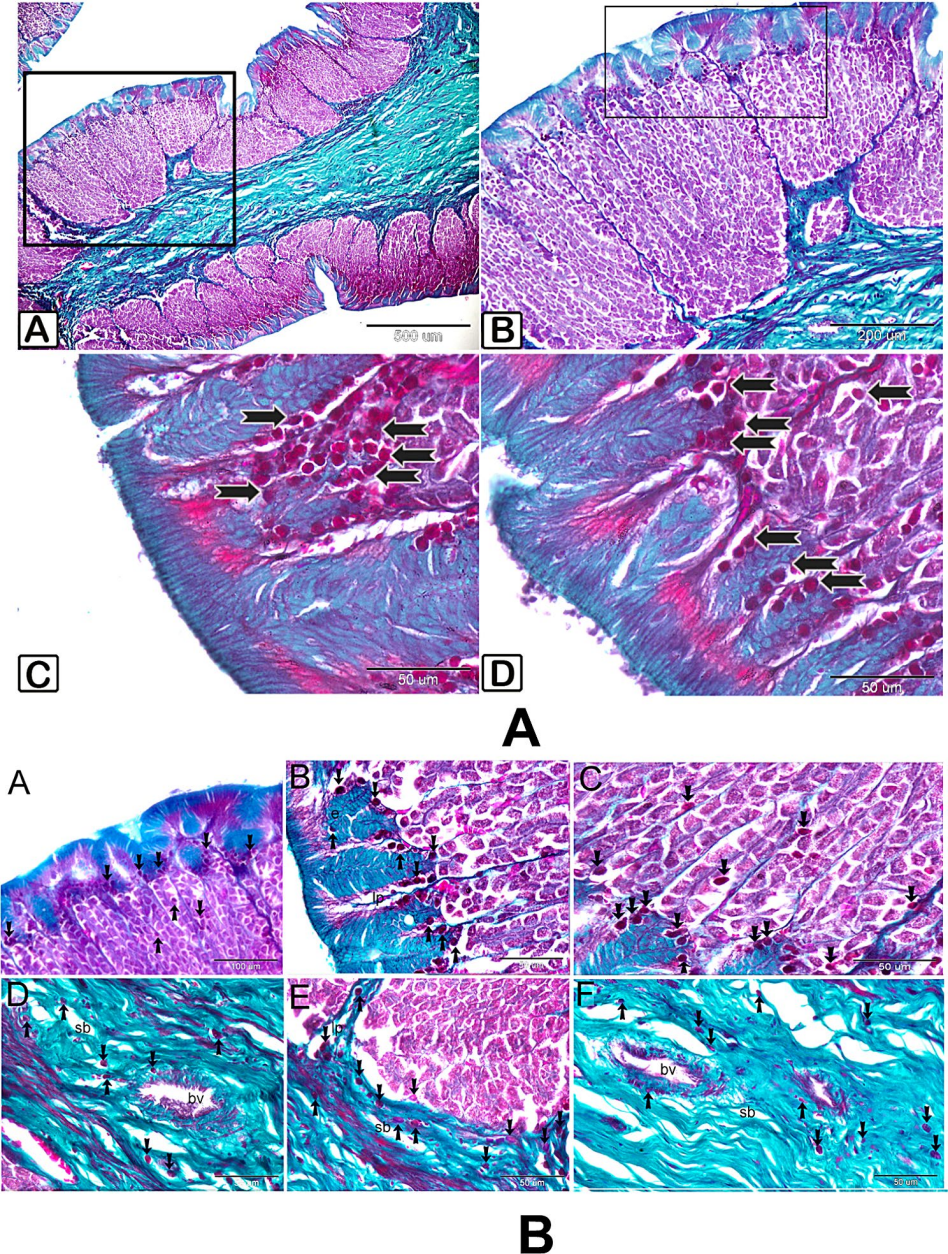
Crossmon’s trichrome histological detection of view of endocrine cell Paraffin cut of the fundic stomach trichrome-stained. Endocrine cells **A** through **D** are found between the gastric gland, and inside the epithelium (arrows). B represents the magnification of a specific area from **A**. **C** and **D** are higher magnifications of selected areas from figure **B**. Endocrine cells **A** through **C** are found between the gastric gland (gl), in the lamina properia (lp), and inside the epithelium (e). D–F: Submucosa (sb) and lamina properia (lp) endocrine cells. Remark blood vessel (bv)

Using Crossmon’s trichrome, endocrine cells were dyed red (Fig. 2a and b). Figure 2a and b(A_C) show the gastric gland and its surrounding submucosa, whereas Fig. 2b(D_F) show the location of the endocrine cells at the deeper part of the epithelium and the lamina propria.

**Fig. 3:**
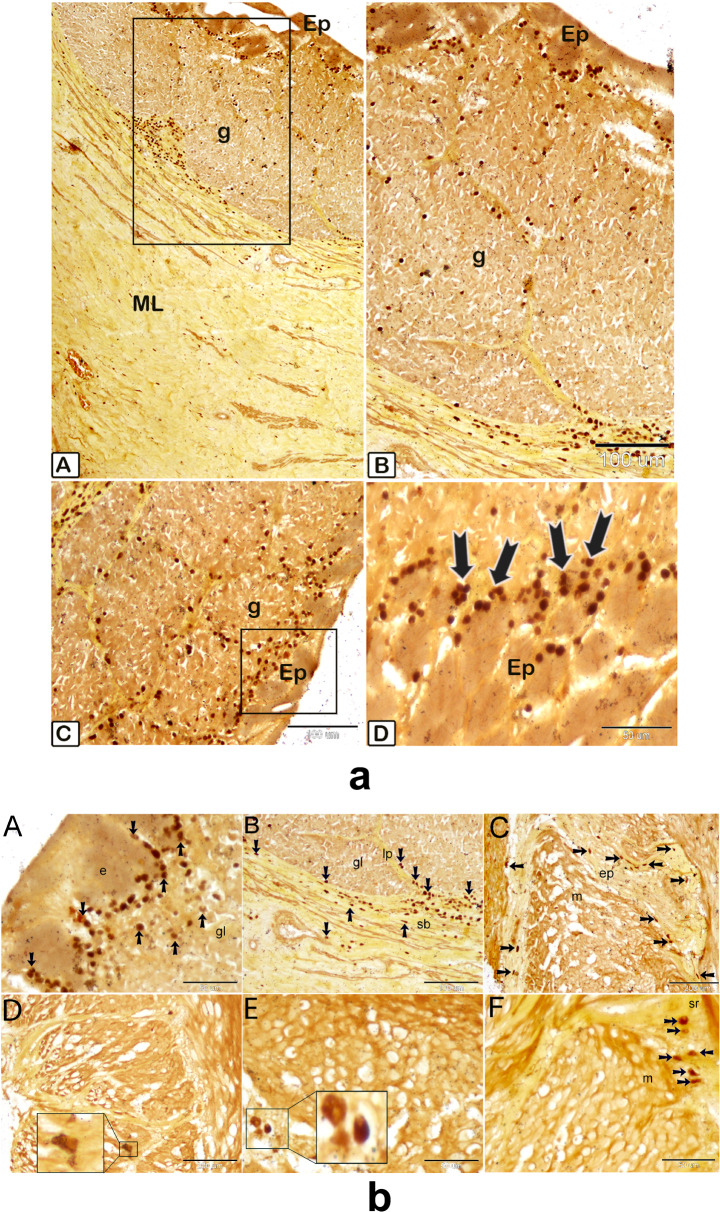
illustrates the general view of arrangement of endocrine cells throughout the lining of the fundic stomach. The endocrine cells (shown by arrows) are situated within the epithelium (EP), muscle layer (ML)and within the gland(g). illustrates the arrangement of endocrine cells throughout the lining of the fundic stomach. A paraffin cut of the fundic stomach was stained using a silver stain. **A**: Endocrine cells, shown by arrows, are clustered beneath the epithelial layer and within the stomach gland. Observe the epithelium (e). **B**: Endocrine cells, indicated by arrows, are clustered in the lamina propria (lp) and submucosa (sb). Endocrine cells (shown by arrows) are clustered together in the epimysium (ep). Observe the muscles (m). The endocrine cells (shown by arrows) are situated within the muscle layer (m). F: Clusters of endocrine cells (shown by arrows) gathered in the serosa (sr)

Using silver stain, the dispersion of endocrine cells could be clearly detected (Fig. 3A). The gastric gland, the epimysium enclosing the stomach’s muscular coat, the lamina propria and submucosa, the serosa, and the subepithelial spaces were all areas where endocrine cells gathered (Fig. 3B: 3A, 3B, 3 C, 3D, 3E, 3 F). The presence of isolated endocrine cells within the myofibrils of muscles (3B: B, D, E and F).

**Fig. 4:**
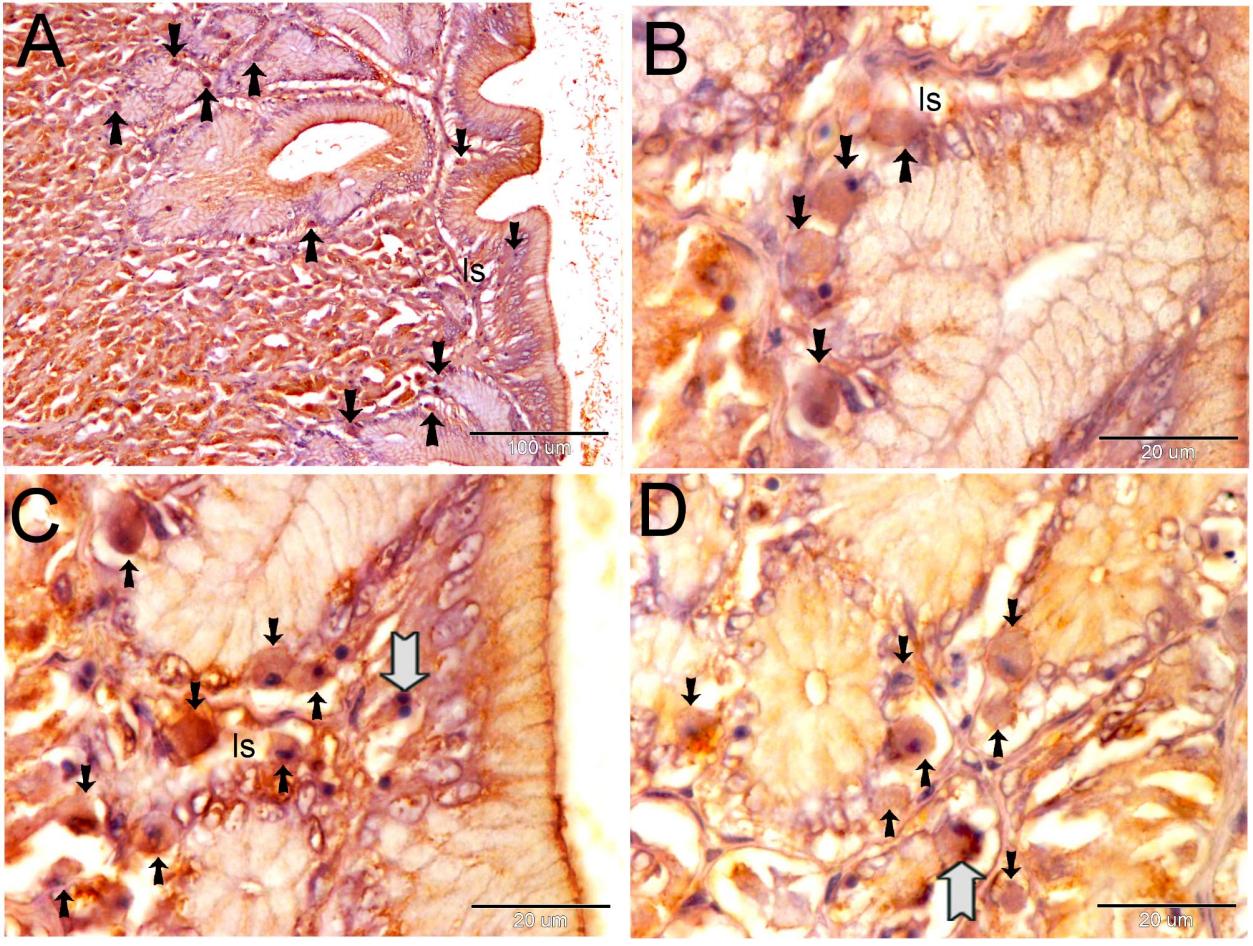
Shows the immune reactivity of the endocrine cells with CD3. The fundic stomach paraffin section was immune stained with CD3. CD3-positive endocrine cells (shown by arrows) formed clusters in the sub-epithelial lymph area. Observe the presence of endocrine cells with two nuclei (shown by red arrows)

**Fig. 5:**
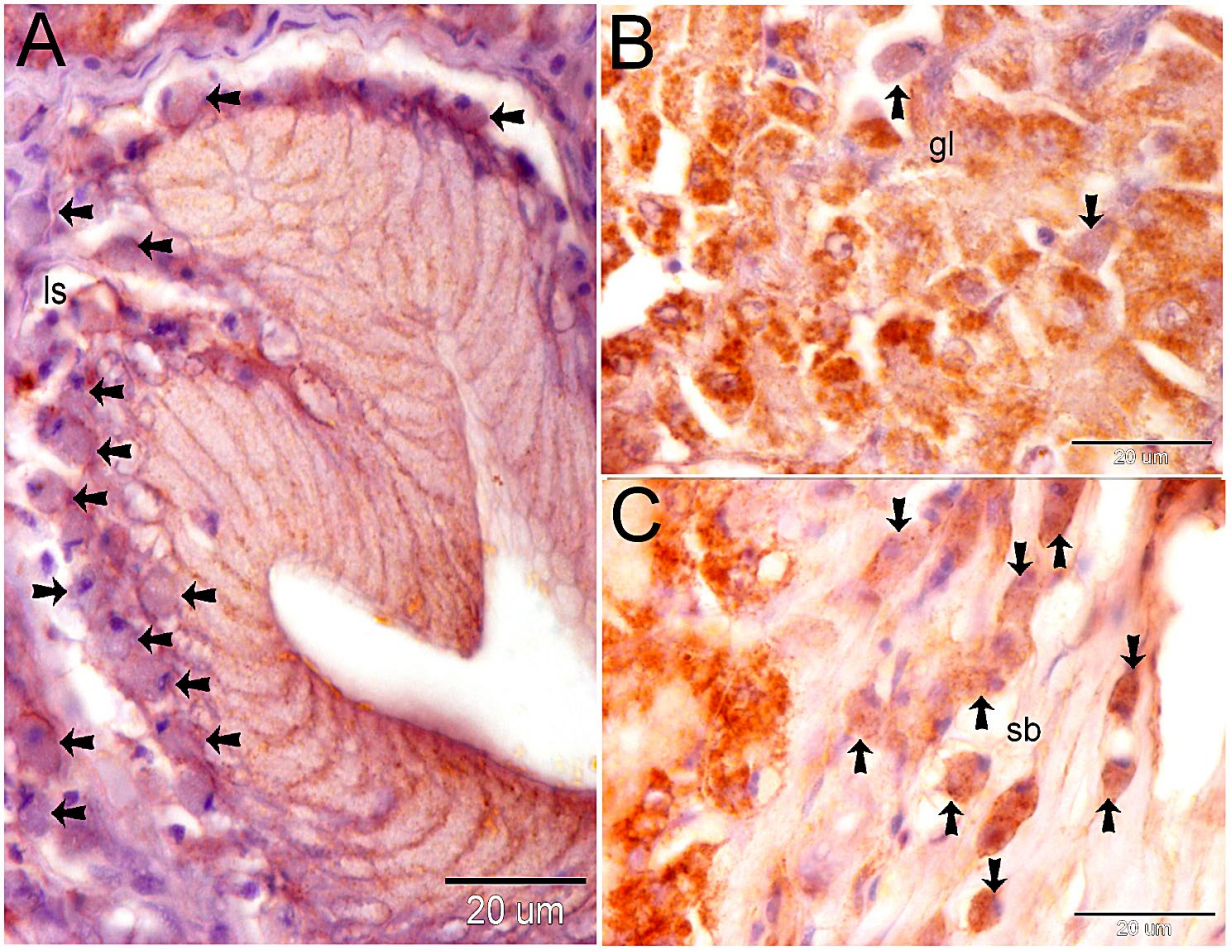
Shows the immune reactivity of the endocrine cells with CD21. The fundic stomach paraffin section was immune stained with CD21. A: Endocrine cells expressing CD21 (shown by arrows) are clustered in the sub-epithelial lymphatic space (ls). B: Endocrine cells positive for CD21 (shown by arrows) are found within the stomach gland (gl). The submucosa contains endocrine cells (CD21-positive) that are shown by arrows

Endocrinology cells react immunohistochemistry with CD3, CD21, and CD68. The subepithelial lymph space was filled with CD3-positive endocrine cells. We also found endocrine cells with multiple nuclei (Fig. 4A-D). The endocrine system secretes CD21 and CD68, which are unique markers of phagocytic cells. There were a lot of CD21 + endocrine cells in the subepithelial lymph space (Fig. 5A), the gastric gland (Fig. 5B), and the submucosa (Fig. 5C).

**Fig. 6:**
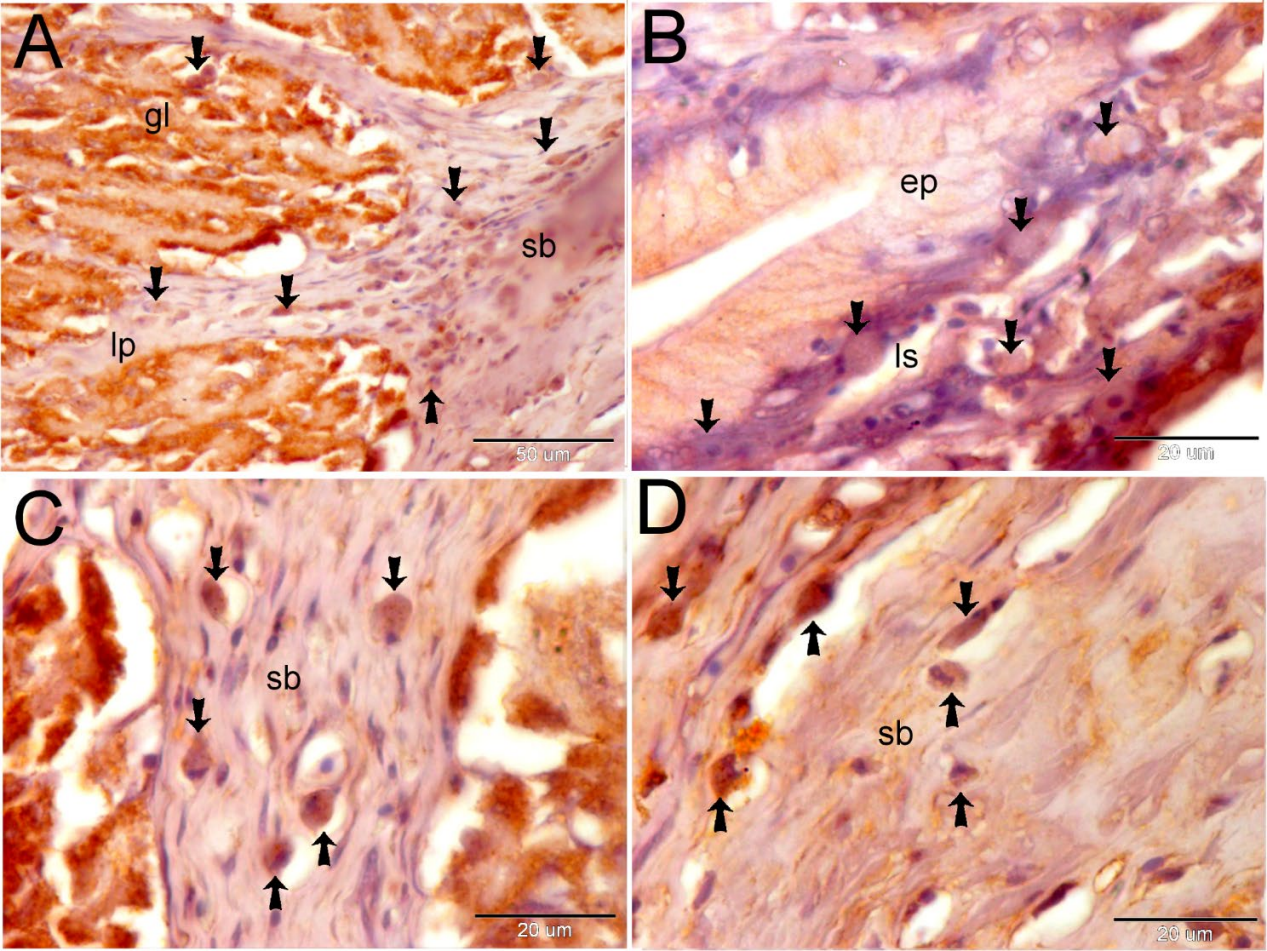
Shows the immune reactivity of the endocrine cells with CD68. The fundic stomach paraffin section was immune stained with CD68. **A**: Endocrine cells positive for CD68 (shown by arrows) are seen in the gastric gland (gl), lamina propria (lp), and submucosa (sb). **B**: Endocrine cells positive for CD68 (shown by arrows) formed clusters within the sub-epithelial lymphatic space (ls). **C**, **D**: CD68-positive endocrine cells (shown by arrows) are situated in the submucosa (sb)

CD68-positive endocrine cells are seen in the submucosa, lamina propria, and stomach gland (Fig. 6A). Endocrine cells positive for CD68 gathered in clumps in the submucosa and subepithelial lymph spaces (Fig. 6C, D).

**Fig. 7:**
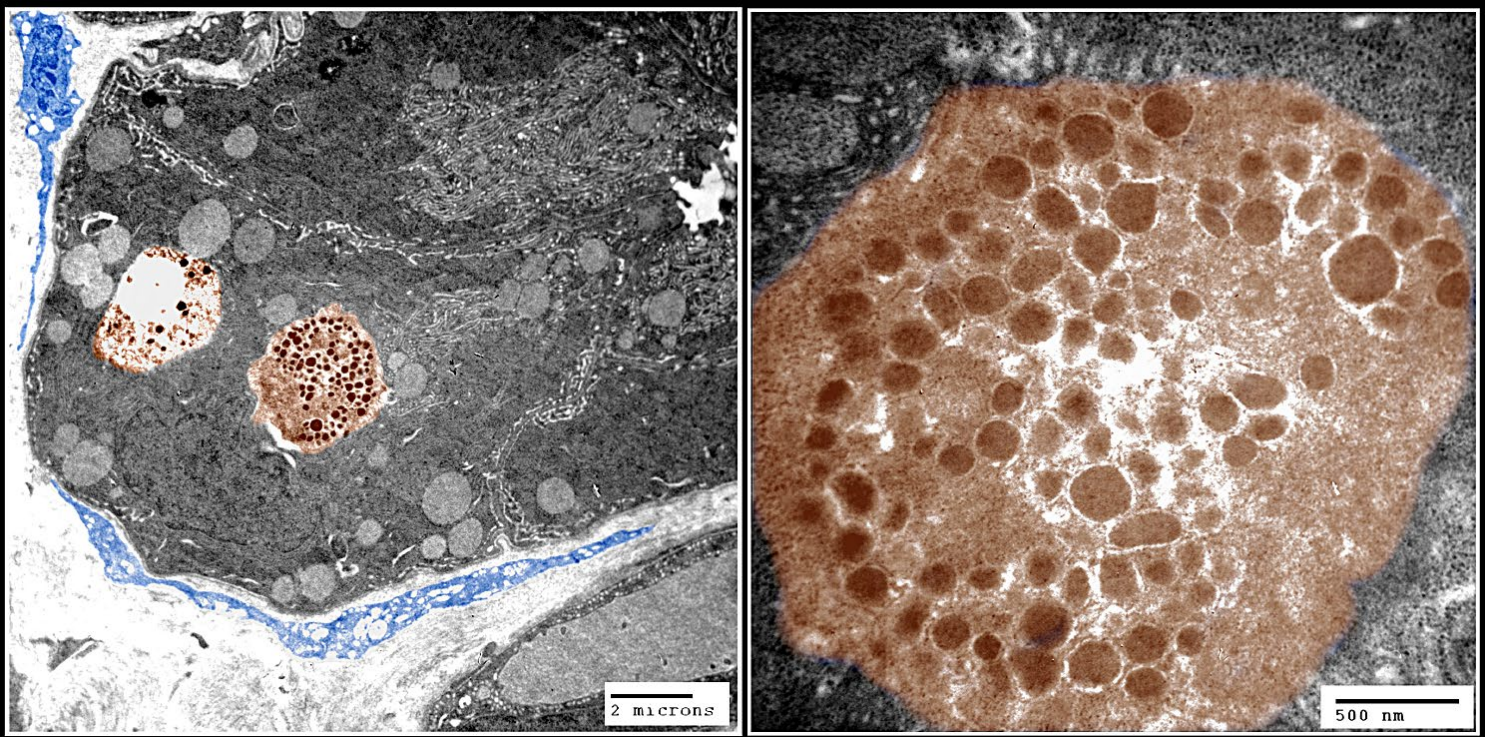
Transmission of digital coloring Under the fundic gland’s epithelium, electron micrographs reveal the ultrastructure of endocrine cells, which are brown in color. Granules of varying sizes and electron densities are densely packed into the cytoplasm. The blue cells around the gland are telocytes

Transmission electron microscopy provided convincing evidence of the presence of cells in the glandular epithelium. Endocrine cells are distinguished by the presence of prominent dense-core granules within their cytoplasm (Fig. 7).

**Fig. 8:**
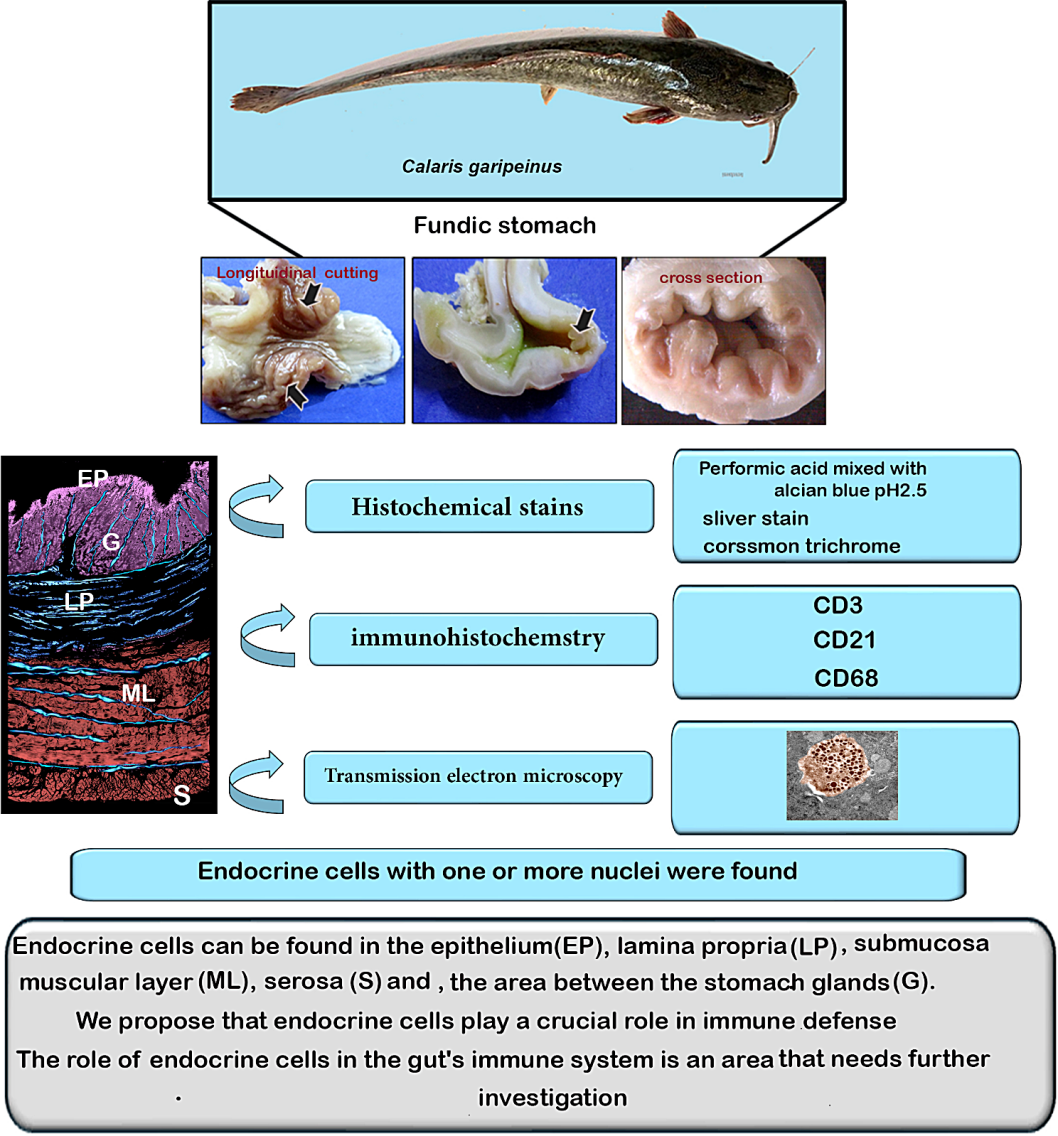
Summarizes the results and conclusions

All negative figures are presented in supplemental files. Figure 8 summarizes the Results and Conclusions.

## Discussion

The endocrine pathway or paracrine signals allow enteroendocrine cells to release hormones into the bloodstream, which is how they function. The gut is a typical place for intraepithelial endocrine cells to be found [25].

The immune system is significantly influenced by endocrine cells, particularly those located in the gastrointestinal tract, through a variety of mechanisms. They are essential players in the maintenance of immune homeostasis and the response to pathogenic threats due to their capacity to secrete hormones and peptides, Ghrelin: Stomach-produced ghrelin reduces inflammation and regulates T and macrophage activity [26]. Gastrin impacts gut-associated lymphoid tissue (GALT), immune cell proliferation and development [27]. Enteroendocrine cells generate defensins, which kill infections and influence immune responses [28]. Serotonin affects dendritic cells and macrophages, modulating the immunological response [29]. Interaction with Immune Cells [30]. Interact directly with immune cells, Enteroendocrine-Immune Cell Crosstalk: Enteroendocrine cells secrete cytokines to recruit and activate intestinal mucosal immune cells like lymphocytes and macrophages [31]. Enteroendocrine cells give antigens to immune cells to start immunological responses [32], modulate the gut microbiota, Hormone and peptide production by enteroendocrine cells affects gut microbiota composition and function, which affects immune responses [33]. regulate inflammation, and Anti-inflammatory effects: Enteroendocrine cells produce anti-inflammatory cytokines and peptides such somatostatin, which inhibits pro-inflammatory cytokines [34]. Pro-inflammatory Responses: Enteroendocrine cells may produce hormones that boost immune defenses during infections [35]. maintain epithelial barrier integrity, Enteroendocrine cells generate hormones and peptides that maintain the gut epithelial barrier, preventing pathogen and toxin translocation into the bloodstream [36]. Repair and Regeneration: Enteroendocrine cells release substances that repair and regenerate epithelial lining after injury or infection, maintaining barrier function. Interactions between neurons and immune cells [37]. The enteric nervous system (ENS) regulates immunological responses via neurotransmitters from enteroendocrine cells. and engage in neuro-immune interactions [38]. Further comprehension of these mechanisms could provide novel insights into therapeutic strategies for gastrointestinal diseases and immune-related disorders.

The location of endocrine cells within the organ’s layers, both intraepithelial and interstitial, and their distribution throughout the organ are noteworthy. It is possible that these aids paracrine signaling instead of endocrine signaling. Mononucleated cells are normal presentation for gut enteroendocrine cells [39]. On the other hand, we found endocrine cells with a binucleated or multinucleated shape. There has been prior documentation of binucleated endocrine cells in rabbit vagina [40]. Found in the lymphatic spaces within the epithelial cells are aggregations of endocrine cells. Based on these findings, it appears that endocrine cells engage in lymphatic migration. The rabbit vagina also showed a similar pattern of behavior. In close proximity to lymphatic and blood vessels, the author discovered massive neuroendocrine cells, which she said migrated along the transcellular pathway and followed lymphatic drainage [40].

The presence of binucleated (double-nucleated) enteroendocrine cells (EECs) across different species is not well-documented in the scientific literature. Binucleated enteroendocrine cells appear throughout species, and their mechanisms can be understood by studying cellular differentiation and reproductive decision-making. Rats on low-sodium diets had binucleate cells in the adrenal cortex’s glomerular zone, showing that special environmental conditions can cause amitotic nuclear division [41]. This suggests that binucleation may be a reaction to physiological or nutritional stressors. Transcription factors and regulatory DNA elements control cell-specific gene expression in enteroendocrine cells, which differentiate into enterocytes, goblet cells, paneth cells, and enteroendocrine cells [42]. The intestinal neuroendocrine subpopulations’-controlled gene expression and geographical distribution may affect binucleation. Reproductive decisions are also influenced by the social decision-making network (SDMN) and neuroendocrine signaling pathways, which may indirectly alter cellular differentiation and binucleated cell predominance [43]. Despite the lack of direct evidence, environmental factors, dietary conditions, and tightly regulated differentiation processes suggest that binucleated enteroendocrine cells may be a common adaptive response in various species. Comparative research across species is needed to measure prevalence and understand the processes behind this phenomenon. While CD3, CD21, and CD68, were used to investigate the immunohistochemical characteristics of endocrine cells. The strong immunoreactivity of the endocrine cells for CD3 is surprising. A specific indicator for T cells is the CD3 marker [44].

Endocrine cells also exhibited markers associated with phagocytic cells, such as CD21 and CD68. Complement receptor 2, also known as CD21, is a naturally occurring molecule that binds to complement component C3 fragments, the low-affinity immunoglobulin (Ig)E receptor CD23, and the type I cytokine interferon-alpha. CD21 is a crucial element of stimulated B- and T-lymphocytes. CD21 interacts with two intrinsic immunological receptors: DNA-DNA complexes (chromatin) and interferon, a cytokine with antiviral properties [45].

CD68, a member of the D scavenger receptor family, and is expressed in conjunction with the formation of late endosomes and lysosomes. Phagocytes that have CD68 in their granules include macrophages, osteoclasts found in bone, microglial cells in the brain, Kupffer cells in the liver, and Hofbauer cells in the placenta [46].

Other immune cell types that express CD68 include lymphocytes, dendritic cells, mast cells, neutrophils, and basophils, among others [47]. The activation of hormones necessitates the lysosomal activity of endocrine cells [48].

Overall, the presence of CD3, CD21, and CD68 in endocrine cells, as well as their widespread distribution across the entire stomach layer, indicates that endocrine cells play a role in immune defense. Subsequent research should investigate the function of endocrine cells in the immune system of the gastrointestinal tract.

## Electronic supplementary material

Below is the link to the electronic supplementary material.

Supplementary Material 1

## Data Availability

All data obtained is included in this manuscript and is available on request from the corresponding authors. There is no sequence data in this manuscript.
