# Supplementary material for: Characterization of giant endocrine cells in the fundic stomach of African catfish (Clarias gariepinus) demonstrated by histochemical, immunohistochemical and ultrastructure microscopy methods suggesting their role in immunity
Source: BMC Vet Res. 2024 Sep 14;20:415. doi: 10.1186/s12917-024-04237-y (PMC11401327; doi:10.1186/s12917-024-04237-y)
Supplement: Supplementary file 1 — Supplementary Material 1 [file 12917_2024_4237_MOESM1_ESM.docx]

**Characterization of giant endocrine cells in the fundic stomach of African catfish (Clarias gariepinus) demonstrated by histochemical, immunohistochemical and ultrastructure microscopy methods suggesting their role in immunity**

**Hanan H. Abd-El-Hafeez* 1, Zyad M. Baker2, Mohamed Aref3, Mohamed A.M. Alsafy4, Samir A.A. El-Gendy4, Eman Zahran 5 , Hams M.A. Mohamed6, Ali H. Alghamdi7, Mahmoud Osman Khalifa8, Sulaiman Mohammed Alnasser9, Basma M. Kamal 10 , Fawzyah A. Alghamdi11, Soha A. Soliman*12, Diaa Massoud13**

1 Professor of cell and tissue, Department of Anatomy and Histology, Faculty of Veterinary Medicine, Assiut University, Assiut (71516), Egypt, Orchid: https://orcid.org/0000-0002-2547-0709, hhnnzz91@aun.edu.eg

2Faculty of medicine, Assiut university, Ziad.Mohammed3778@med.aun.edu.eg

3 Department of Anatomy and Embryology, Faculty of Veterinary Medicine, Zagazig University, Zagazig 44519, Egypt. abdelazizanatomy11@gmail.com

4Anatomy and Embryology Department, Faculty of Veterinary Medicine, Alexandria University, Abis 10th P.O. 21944, Alexandria, Egypt.

Samir A.A. El-Gendy, Samir.algendy@alexu.edu.eg

Mohamed A.M. Alsafy, mohamed.alsafy@alexu.edu.eg

5 Department of Aquatic Animal Medicine, Faculty of Veterinary Medicine, Mansoura University, Mansoura, Egypt. emanzahran@mans.edu.eg; dr.samiaahmed@mans.ed.eg emanzahran@mans.edu.eg

6 Department of Microbiology, Faculty of Veterinary Medicine, South Valley University, Qena, 83523, Egypt, Drhams85@yahoo.com

7Department of Biology, Faculty of Science, Al-Baha University, Alaqiq, Saudi Arabia.

aayfan@bu.edu.sa, ORCiD: 0000-0003-0426-9237

8Department of Anatomy and Embryology, Faculty of Veterinary Medicine, Aswan University, Aswan, Egypt, mahmoud.othman@aswu.edu.eg

9Department of Pharmacology and Toxicology, College of Pharmacy, Qassim University, Qassim, 51452, Saudi Arabia, sm.alnasser@qu.edu.sa

10Department of Anatomy and Embryology, Faculty of Veterinary Medicine, University of Sadat City, Sadat City 6010230, Egypt, basma.shehata@vet.usc.edu

11Department of biological science, College of Science, University of Jeddah, Jeddah, Saudi Arabia, P.O. Box 80327, Jeddah 21589.

12Department of Histology, Faculty of Veterinary Medicine, South Valley University, Qena, Egypt. Email: soha_soliman@vet.svu.edu.eg

13Department of Zoology, Faculty of Science, Fayoum University, Fayoum, Egypt. msm07@fayoum.edu.eg


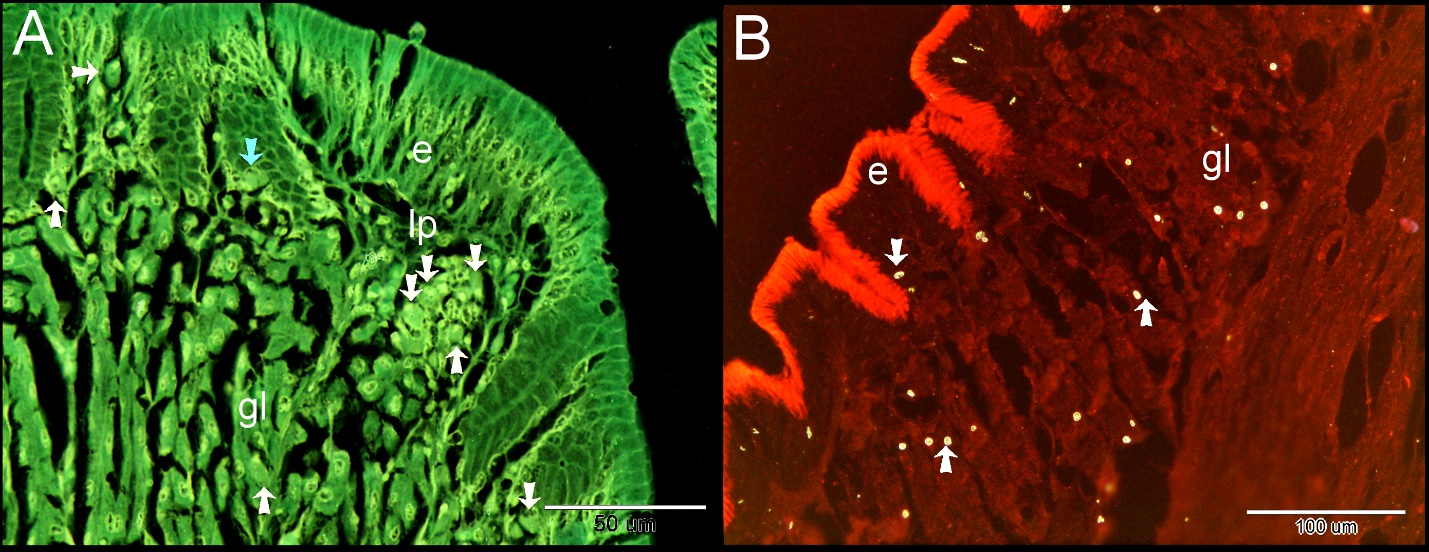


Supplementary figure 1: Negative of figure 1.


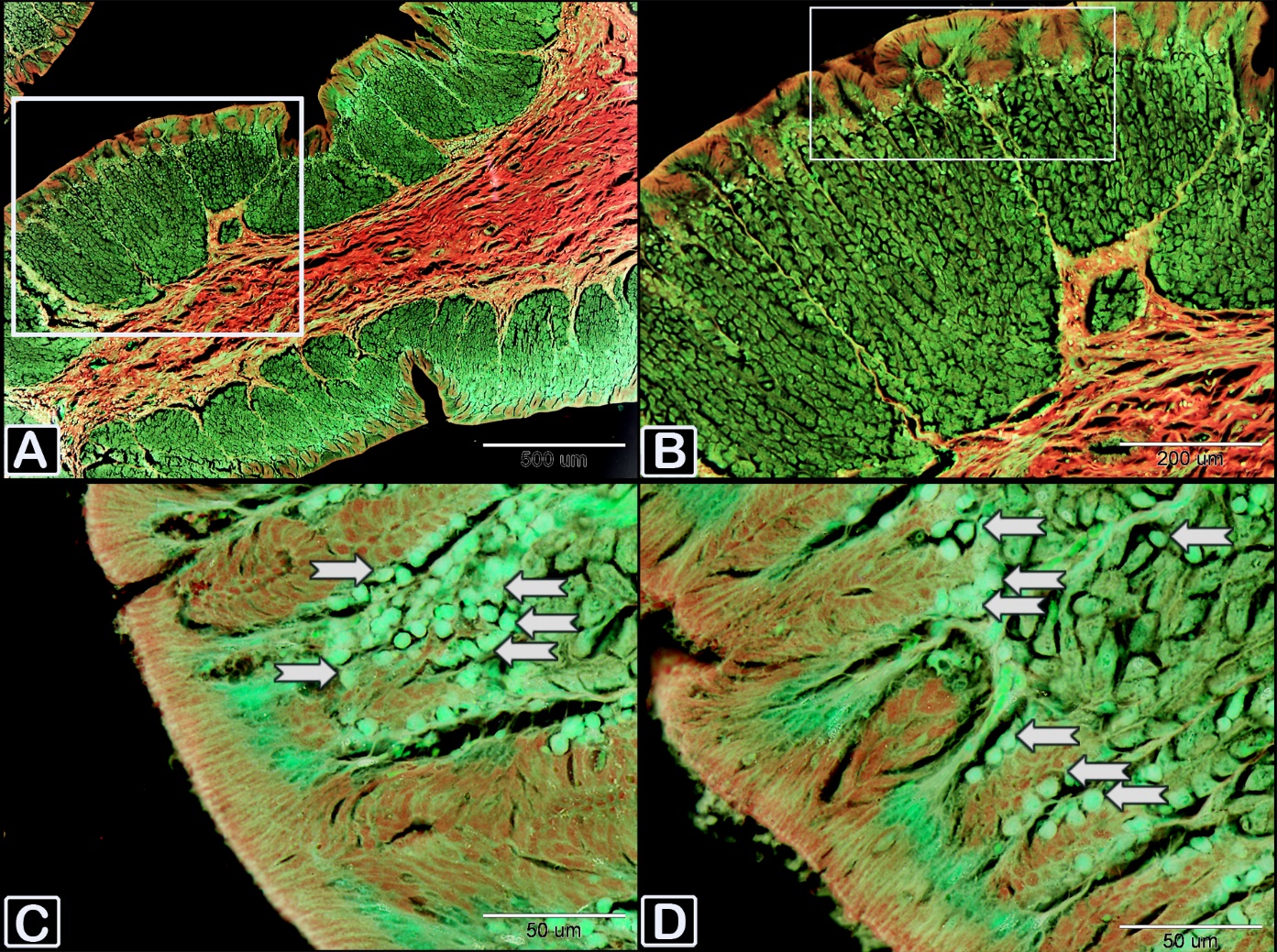


Supplementary figure2: Negative of figure 2a.


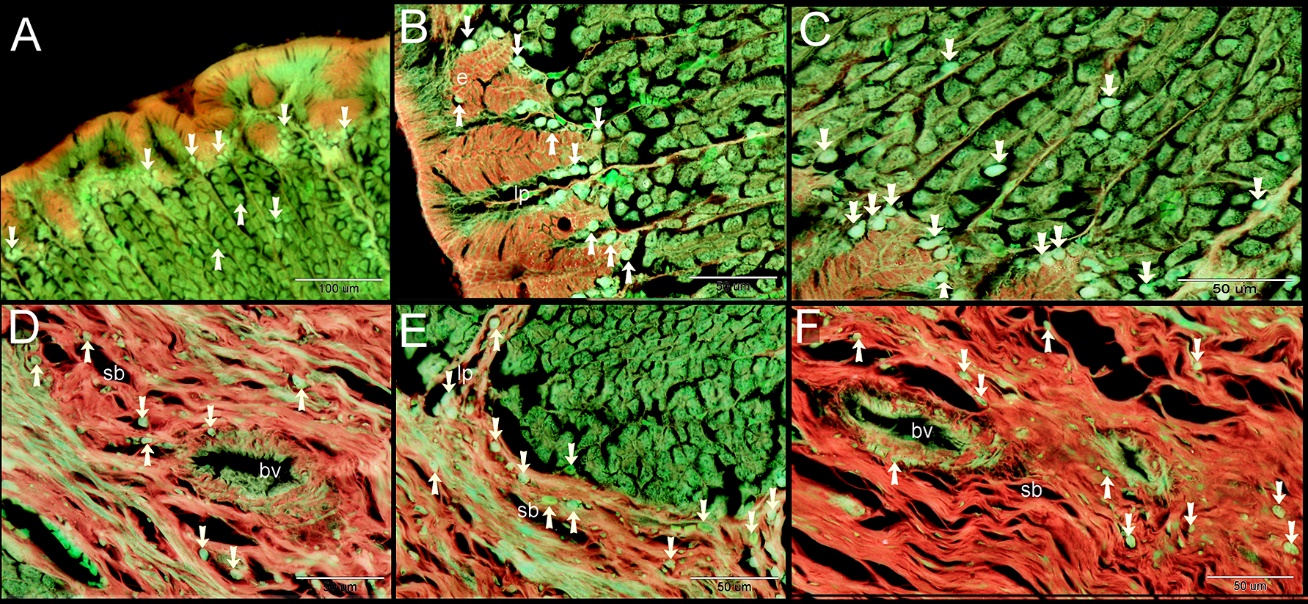


Supplementary figure 3: Negative of figure 2b.


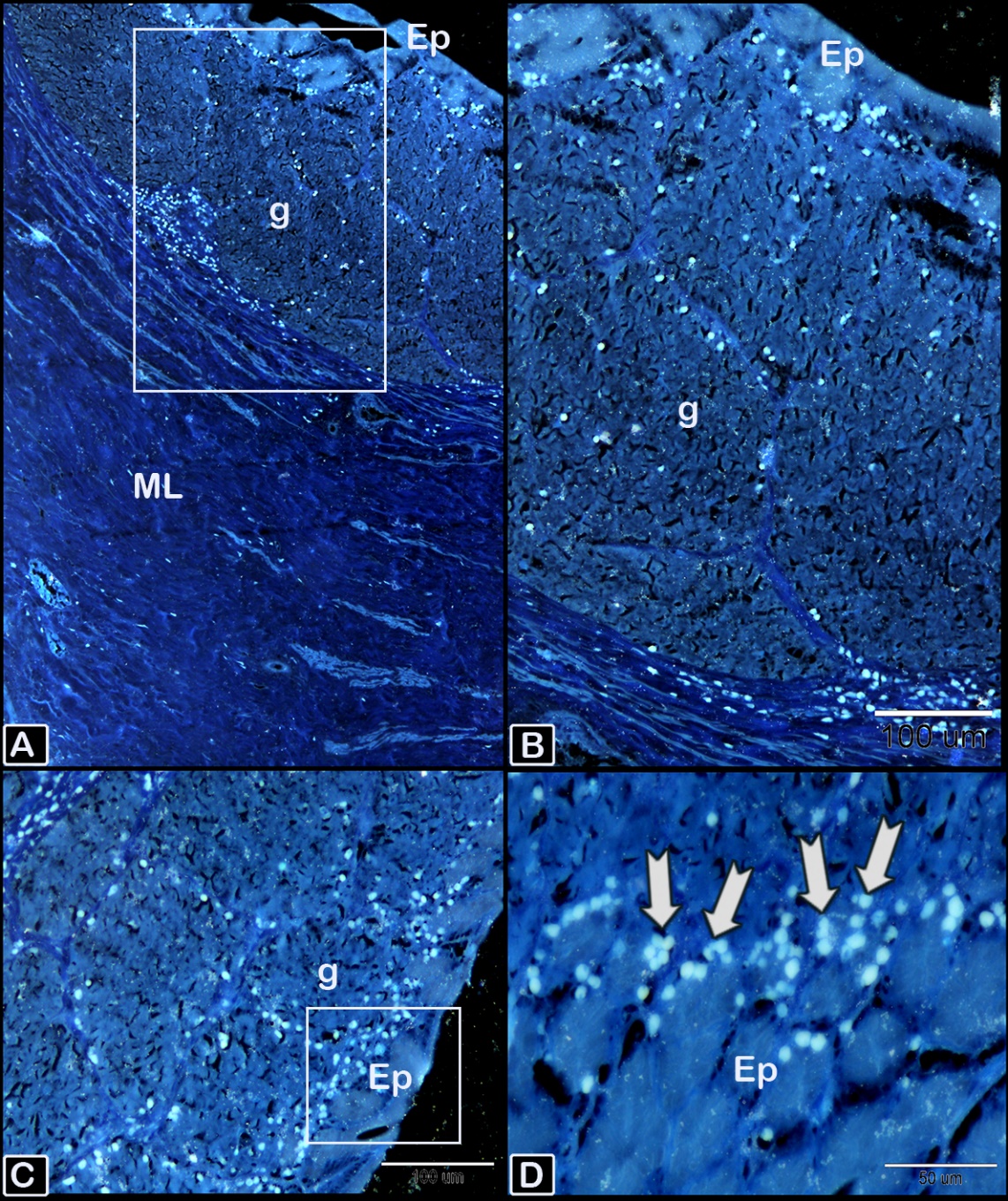


Supplementary figure 4: Negative of figure 3a.


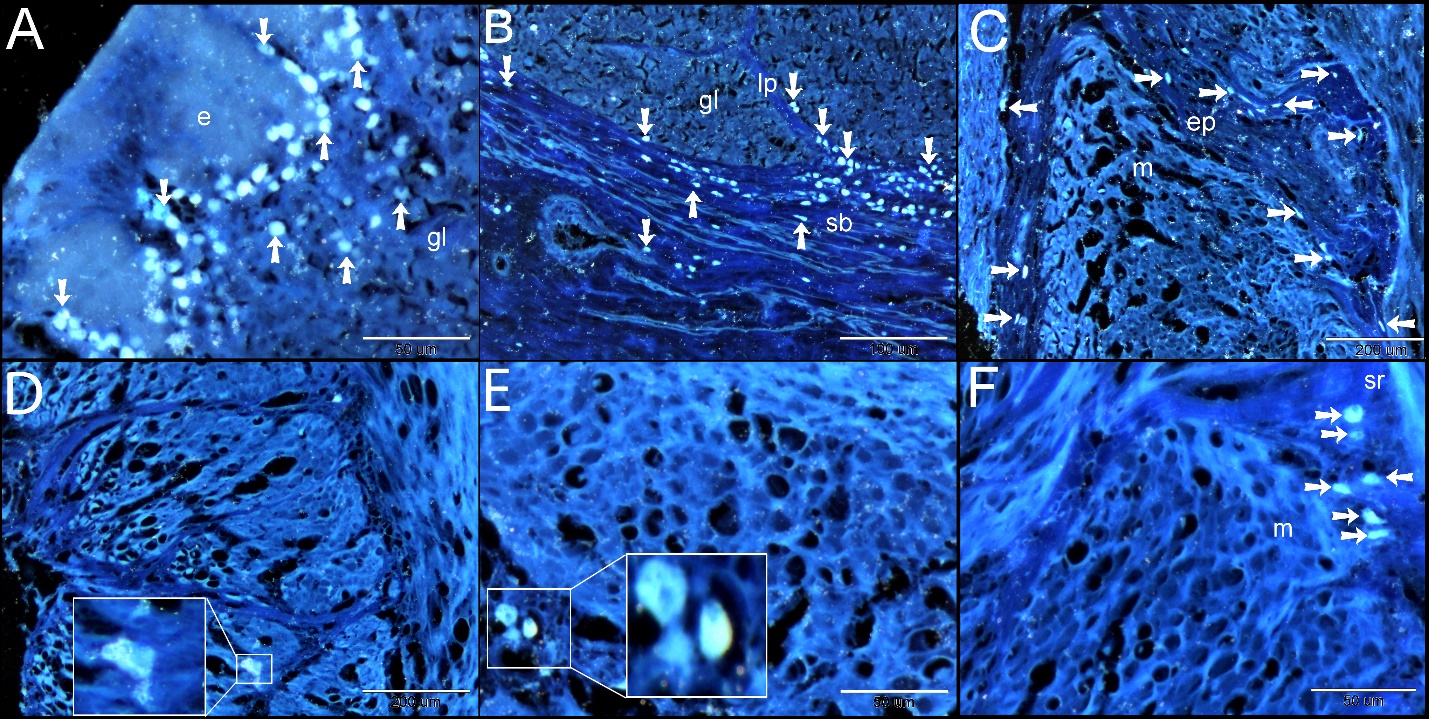


Supplementary figure5: Negative of figure 3b.


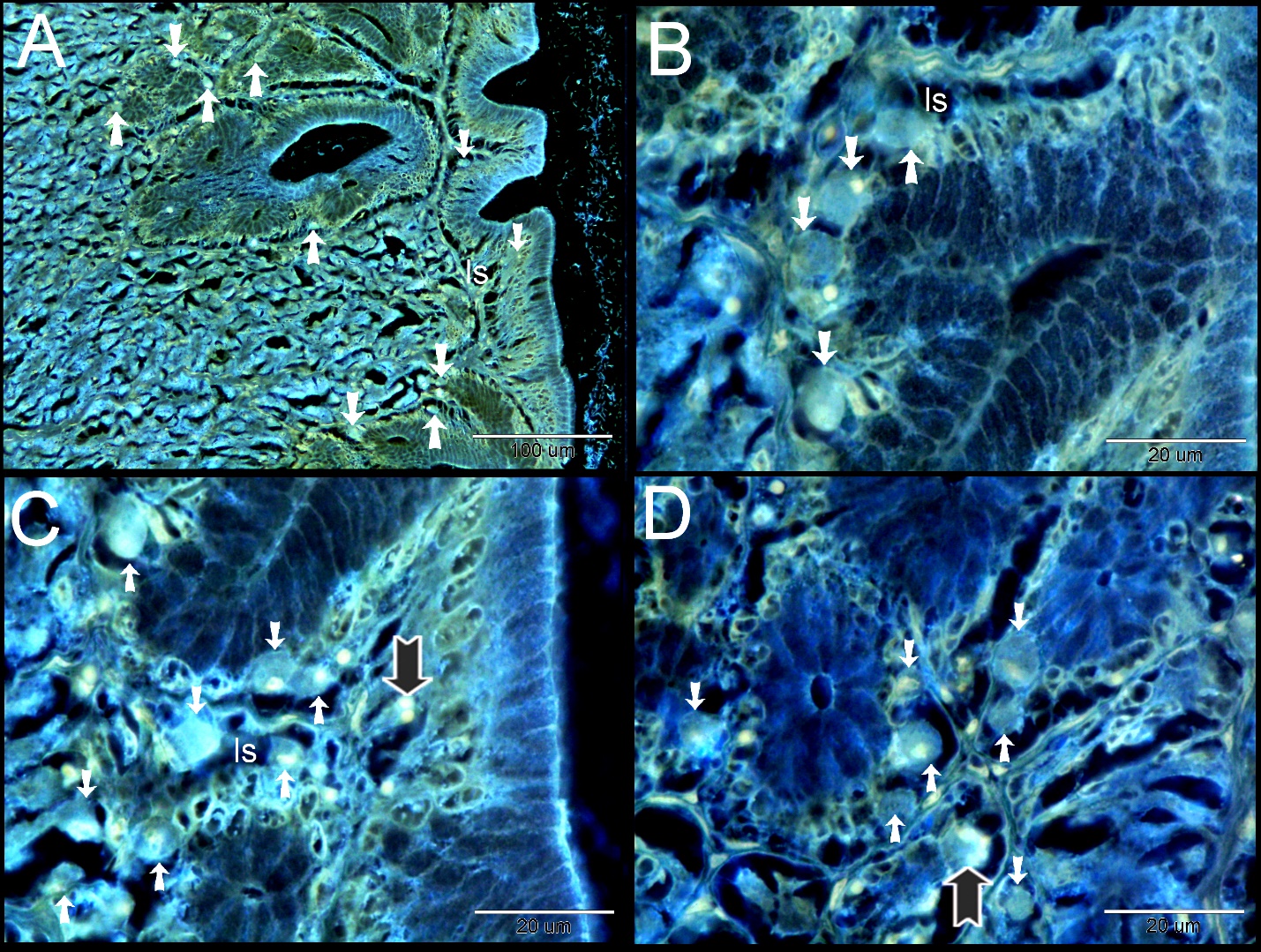


Supplementary figure 6: Negative of figure 4.


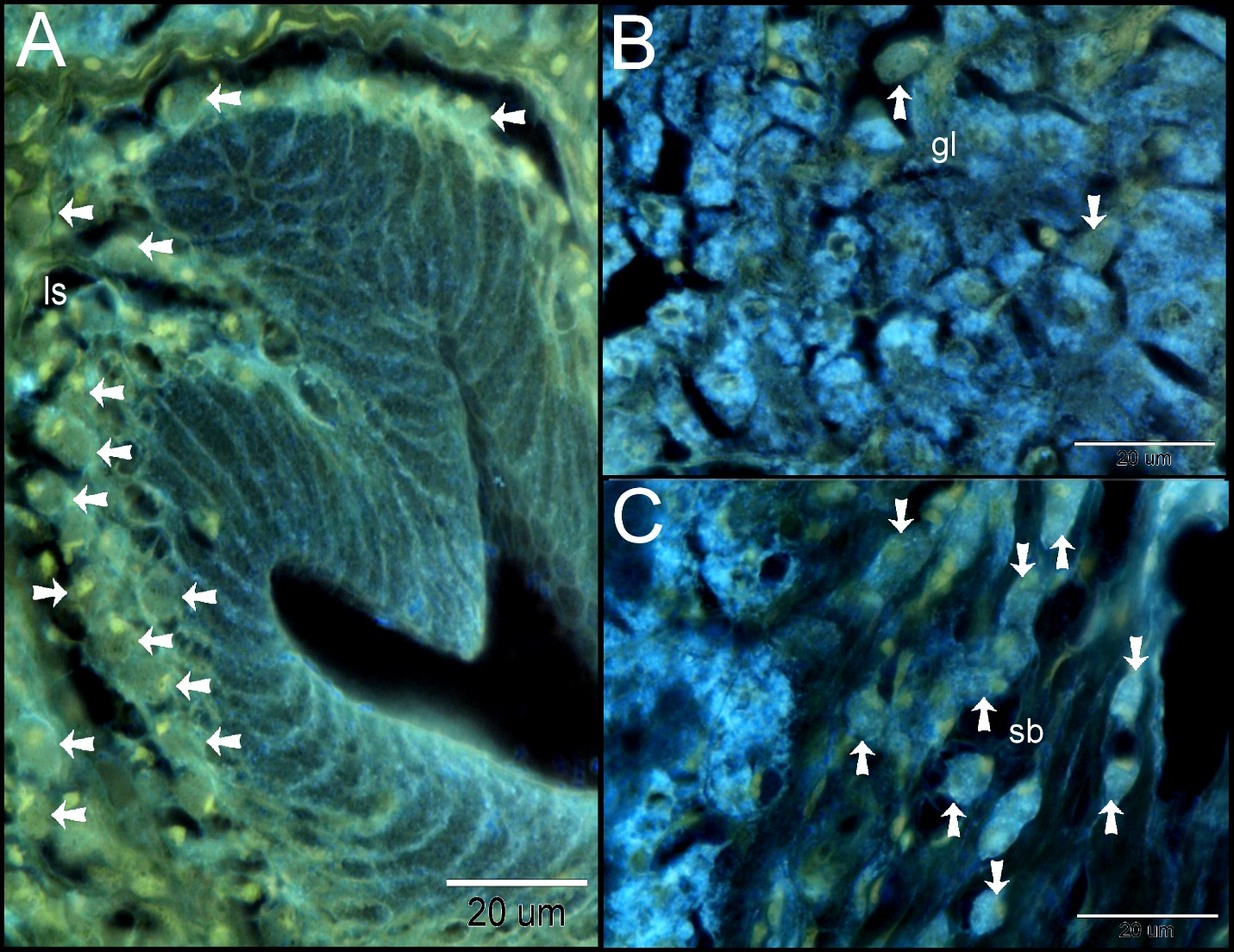


Supplementary figure 7: Negative of figure 5.


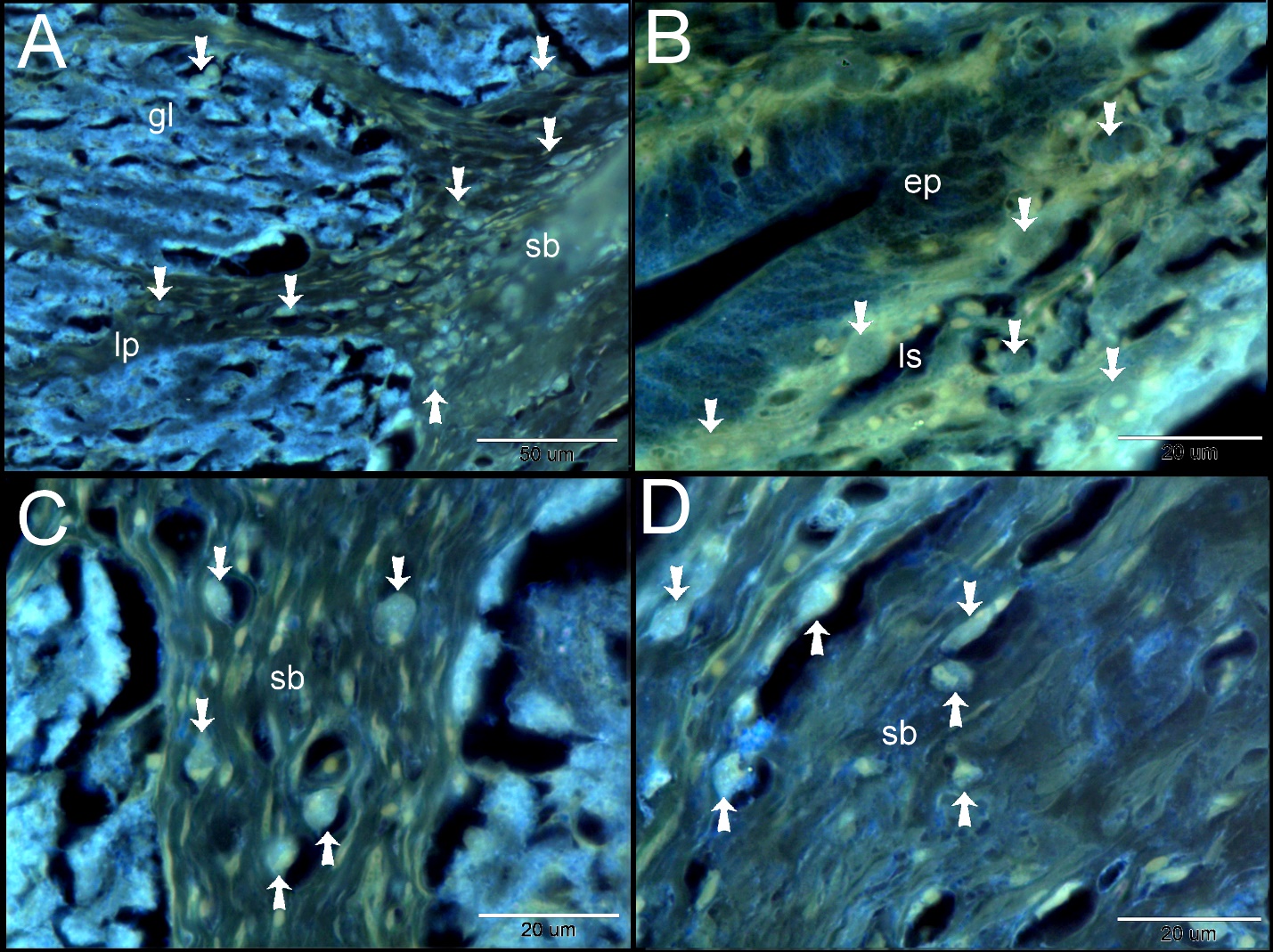


Supplementary figure 7: Negative of figure 6.


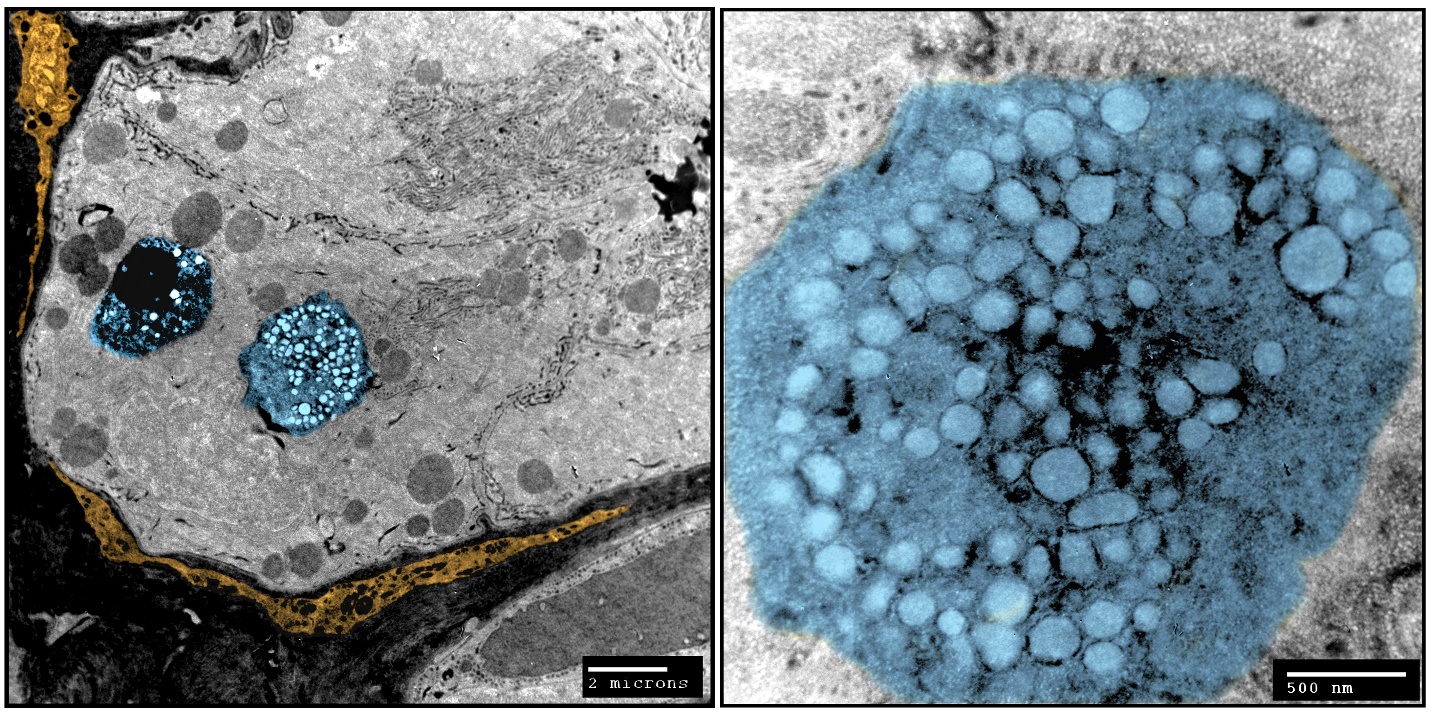


Supplementary figure 8: Negative of figure 7.
